# Behavioral Interventions Improve Mailed Colorectal Cancer Screening Among Overdue Patients in a Randomized Trial

**DOI:** 10.1016/j.cgh.2025.09.015

**Published:** 2025-09-20

**Authors:** Shivan J. Mehta, Pranay Nadella, Pamela A. Shaw, Catherine Reitz, Caitlin Brophy, Evelyn Okorie, Keyirah Williams, Christopher K. Snider, MaryAnne K. Peifer, Corinne Rhodes, David A. Asch

**Affiliations:** 1 Perelman School of Medicine, University of Pennsylvania, Philadelphia, Pennsylvania; 2 Center for Health Care Transformation and Innovation, University of Pennsylvania, Philadelphia, Pennsylvania; 3 Biostatistics Division, Kaiser Permanente Washington Health Research Institute, Seattle, Washington

**Keywords:** Behavioral Economics, Colorectal Cancer Screening, Outreach, Reminders, Text Messaging

## Abstract

**BACKGROUND & AIMS:**

Mailed fecal immunochemical testing (FIT) can boost colorectal cancer screening (CRC) rates, but response rates remain limited. We evaluated if behavioral interventions increased response rates.

**METHODS:**

This pragmatic randomized trial with a 2 × 2 × 2 factorial design included primary care patients aged 50 to 74 years who were overdue for CRC screening. Patients were randomized 1:1 to receive a health system-branded blue box or a standard envelope. Patients were concurrently randomized 1:1 to receive or not receive text reminders. Patients were also concurrently randomized 1:1 to receive a reminder letter. The primary outcome was the proportion who completed a FIT within 4 months.

**RESULTS:**

Among 5244 patients included, the mean age was 59.6 years (standard deviation [SD], 7.3 years); 35.8% were Black and 53% were White. At 4 months, 17.8% (95% confidence interval [CI], 16.3%-19.2%) who received the box, compared with 18.0% (95% CI, 16.5%-19.5%) who received the standard envelope, completed FIT, an absolute difference of −0.2% (95% CI, −2.3 to 1.9%; *P* = .85). Among those who received text messaging, 21.2% (95% CI, 19.6%–22.8%) completed FIT compared with 14.6% (95% CI, 13.2%–15.9%) of those who did not, an absolute difference of 6.6% (95% CI, 4.6%–8.7%; *P* < .001). Among those that received the mailed reminder, 20.3% (95% CI, 18.7%–21.8%) completed FIT compared with 15.5% (95% CI, 14.1%–16.9%) of those who did not, an absolute difference of 4.8% (95% CI, 2.7%–6.9%; *P* < .001).

**CONCLUSIONS:**

Behaviorally informed text messaging and mailed reminders significantly increased screening completion, but the health system-branded box did not increase response rates. ClinicalTrials.gov, Number: NCT05341622.

## Graphical Abstract

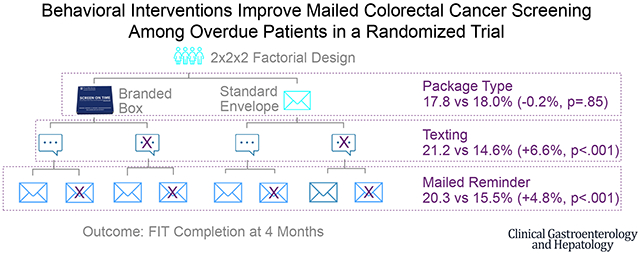

Colorectal cancer (CRC) screening decreases mortality,^1–3^ but a third of United States adults are not up-to-date on screening.^4^ Colonoscopy has been the predominant form of screening in the United States, but strained endoscopy capacity since the COVID-19 pandemic has increased interest in alternative approaches to screening patients at average risk.^5^ An advantage of fecal immunochemical testing (FIT) is that it can be mailed to patient’s homes as part of a population health program.^6^ However, low completion rates to mailed outreach can limit the effectiveness and efficiency of the program.^7^

New insights from the field of behavioral science have shown that short cuts in thinking that often detract from long-term health goals such as present-time bias can be leveraged to improve uptake of preventive health activities,^8–10^ as has been seen in vaccinations and cancer screening.^11–13^ For example, opt-out framing is already incorporated into mailed FIT outreach programs.^14^ Messages delivered by text or letters can prompt patients toward completion, particularly when they include behaviorally informed framing, and prominent packaging for the kit can leverage salience by standing out and attracting attention.^15^ Completion rates improve when messaging comes from trusted sources, such as the primary care clinician,^16,17^ or when norms of reciprocity are introduced as patients are reminded of value received.^18^ Finally, asking people to make plans about intended behaviors ahead of time has been shown to increase preventive health activities through precommitment or implementation intentions.^19–21^

In this pragmatic factorial randomized trial, we simultaneously tested several behaviorally informed strategies with the goal of increasing FIT completion among adults overdue for CRC screening.

## Methods

### Study Design

This was a 2 × 2 × 2 factorial design randomized controlled trial (RCT) designed to assess the impact of the type of packaging used for mailed FIT, text message reminders, and personalized mailed reminders on the rate of FIT completion.

**Figure 1.**
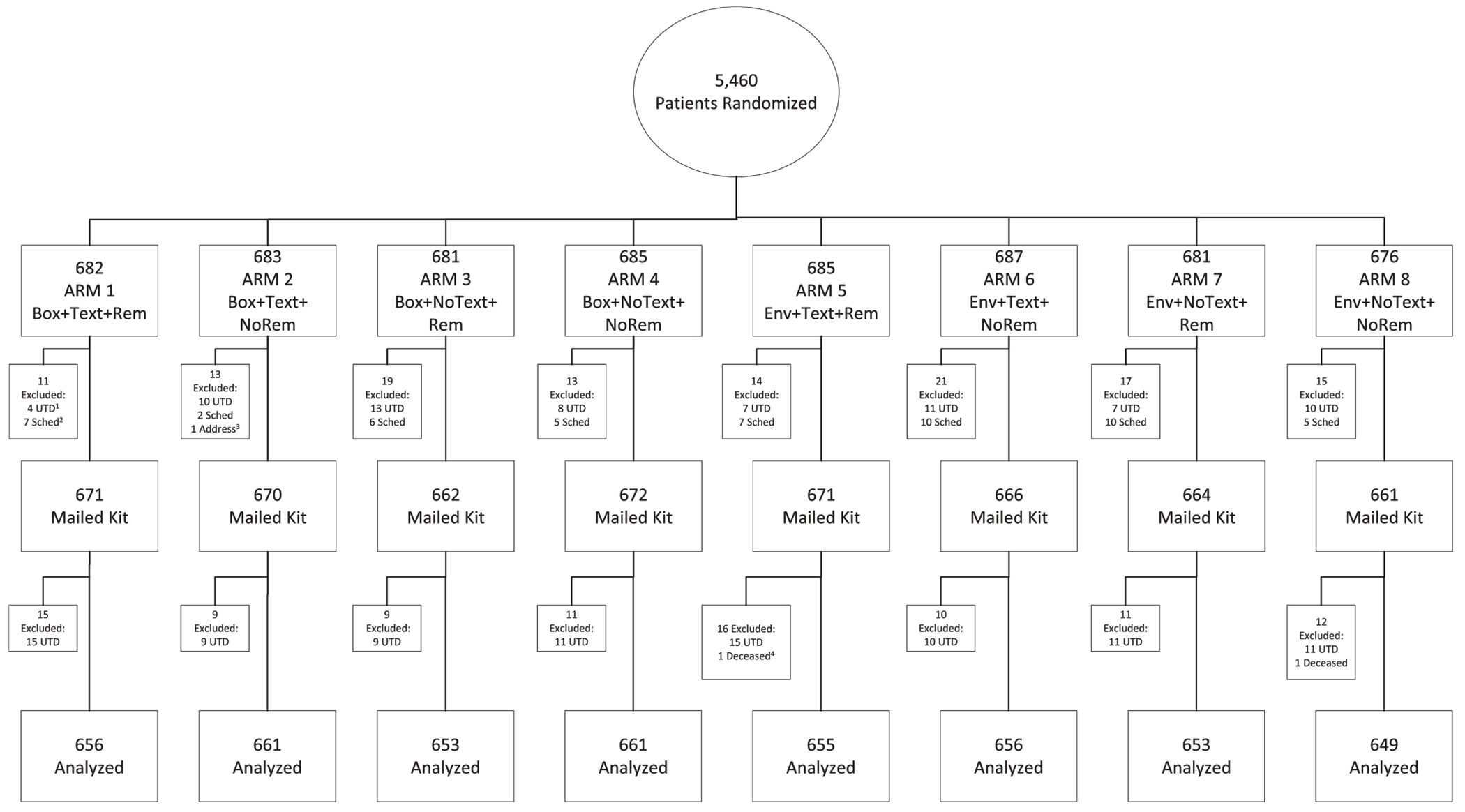
CONSORT diagram. ^1^Up-to-Date (UTD) prior to outreach ^2^Scheduled (Sched) for colonoscopy prior to outreach ^3^Address incomplete/invalid ^4^Died prior to outreach

The study was approved by the Institutional Review Board at the University of Pennsylvania. A waiver of informed consent was granted as the study was minimal risk, was incorporated into existing clinical operations, and would have been impractical to carry out without the waiver.^22^ The protocol and statistical plan appear as a Supplement and the study was registered at ClinicalTrials.gov (NCT05341622). This study followed the Consolidated Standards of Reporting Trials (CONSORT) guidelines (Figure 1). All authors had access to the study data and reviewed and approved the final manuscript.

### Study Population

Using an automated electronic health record (EHR) data extraction in May 2022, we identified patients at average risk for colorectal cancer aged 50 to 74 years who had been seen at least once within the prior 2 years by a primary care provider (PCP) within the University of Pennsylvania Health System (Penn), and who had no colonoscopy in the last 10 years, no stool testing in the last year, no flexible sigmoidoscopy in the last 5 years, and no FIT-DNA in the last 3 years. Patients were excluded if they had personal or significant family history of CRC, or personal history of colonic polyps, hereditary nonpolyposis CRC syndrome, familial adenomatous polyposis syndrome, other gastrointestinal cancer, gastrointestinal bleeding, iron-deficiency anemia, inflammatory bowel disease, history of total colectomy, dementia, or metastatic cancer. Patients were also excluded if they were on hospice or receiving palliative care, were uninsured or self-pay, were scheduled for a colonoscopy or sigmoidoscopy at the time of the study, or had a lab outside the health system documented as their preferred lab for testing.

### Interventions

The study had 3 interventions that were evaluated concurrently in a 2 × 2 × 2 factorial design, with individual randomization in a 1:1 ratio for all comparisons. First, patients were randomized using a computer generated algorithm, stratified by practice, to receive either: (1) a standard mailing envelope containing a letter describing the importance of CRC screening, the patient’s eligibility for FIT, a FIT kit (Polymedco OC-Auto FIT), and information about how to complete and return the test (mail it back in a postage-paid envelope or drop it of at a laboratory); or (2) a health system-branded blue box with additional user-friendly messaging and images to encourage patients to complete and return the test, along with the same FIT kit and instructions.

Patients were concurrently randomized to receive text messaging in addition to mailed outreach. Patients randomized to receive texting were sent a series of behaviorally informed messages about the importance of CRC screening, a recommendation from the PCP (social norms), and provided with an opportunity for feedback. Questions that were submitted via text by patients were manually reviewed and responded to by research staff or routed to the clinician if necessary. The messages invoked reciprocity by reminding them of how the free kits were sent by the PCP and health system, and patients could text back “PLAN” to state that they would return the kit within 7 days, which was a form of precommitment, or implementation intention. Patients also had the option to reply “DONE” if they had already returned their completed kit, “COLO” if they had scheduled or completed a colonoscopy, or “MISSING” if they did not receive their kit. For missing kits, addresses were confirmed and new kits mailed out. For “COLO,” participants were encouraged to share the date, location, and results with their PCP (Supplementary Table 1). Messages were sent prior to FIT mailing, 21 days after FIT mailing, and 56 days after FIT mailing. Text messaging responses concluded 65 days from the date of the first text. The text messaging intervention was conducted using the Way to Health (W2H) platform, a National Institutes of Health-funded software platform that facilitates and automates many aspects of study design and intervention implementation.^23^

Lastly, patients were concurrently randomized, stratified by practice, whether they received the box, and whether they received text messaging, to receive a mailed reminder letter signed by the PCP if they did not respond after 1 month of initial outreach. The letter invoked social norms and reciprocity, and it also provided a time frame to return the kit, as specific deadlines have been shown to increase response rate.^24^ Patients were excluded from all reminder messaging if they completed or were scheduled for a screening procedure or returned a completed FIT.

Patients were notified of their FIT results by their PCP, the standard of care within the practices at the time. FIT-positive patients received additional follow-up from population health coordinators embedded within the practice regions to answer questions and navigate patients toward colonoscopy completion.

Initial outreach for this trial was conducted on a rolling basis between May 27, 2022, and June 10, 2022. The trial concluded December 10, 2022, 6 months from initial outreach. All communications and messaging content are available in Appendix A of the Protocol Supplement.

### Outcomes

The primary outcome of this study was the percentage of patients who completed FIT within 4 months of outreach. Secondary outcomes were completion of any CRC screening at 4 months, completion of FIT at 6 months, and completion of any CRC screening at 6 months. We also tracked the percentage of completed FIT with positive results, the percentage of patients who scheduled and completed colonoscopy, and the results of the procedure (adenoma, advanced adenoma, cancer) if completed.

### Statistical Analysis

Based on our prior work and existing literature,^25,26^ we expected 18% FIT completion in the arm receiving basic outreach (no box, no text message, no reminder), and that each intervention—health system-branded box, text messaging, and mailed reminder—would increase FIT completion by 4%. With 2500 patients in each arm, we estimated we had 90% power to detect a 4% absolute difference for each intervention relative to its control. Our power calculation estimates were based on operational capacity for the processing of FIT, the number of estimated eligible patients and meaningful differences to justify implementation, and costs of each of the single interventions. The factorial design is efficient for studying multiple interventions, resulting in appreciably smaller sample sizes relative to studying each intervention in a separate study.^27^ We did not account for interaction between interventions in the power calculation, as these analyses were planned as exploratory.

For the prespecified primary and secondary analyses, we used χ^2^ tests of 2 proportions and intention-to-treat protocol to assess the difference in FIT completion across an intervention arm and 95% confidence intervals (CIs). Because all patients in the trial received an outreach intervention, we compared the overall CRC screening completion rate among all patients in the trial to patients in similar practices that were not included in the trial (and did not receive mailed FIT outreach) to evaluate the effectiveness of outreach compared with no outreach. Patients in the 17 nonrandomized comparator practices were identified using the same inclusion and exclusion criteria but were not included in outreach due to our lab’s operational capacity to process the kits. We compared completion of any CRC screening method in this group (All Comparator cohort) to all trial participants (All RCT cohort) during the trial followup period. Within one large practice (Bucks), one-half the eligible patients were randomly selected for the trial, and the other one-half could not be included due to operational capacity, so we also compared screening among patients receiving outreach in the trial (Bucks RCT cohort) to those that did not receive outreach (Bucks Comparator cohort).

Race/ethnicity was based on self-reported data in the EHR. Outcome data was extracted from the EHR for this health system and other health systems (CareEvery-where) and was complemented by manual chart review as needed to obtain documented evidence of completion. Household income was estimated using the American Community Survey 2017 to 2021 5-year estimates data for median income by zip code of residence.

We tested for heterogeneous treatment effects using interaction terms between a given subgroup covariate and the treatment arms, using likelihood ratio tests for significance adjusting for all other covariates in the model. All analyses were performed in Stata version 15.0 (Stata Corp LP).

## Results

### Participants

A total of 5460 patients were initially randomized from 17 practices; 216 patients were then excluded; 123 patients were removed from outreach prior to mailing because they were found to be previously up to date on screening or did not have a valid address on file in the EHR. An additional 93 patients were excluded after mailing when found to be up to date on screening or deceased prior to outreach. The analytic sample of 5244 patients received mailed FIT and were included in our analysis (Figure 1). The mean age was 59.6 years (standard deviation, 7.3 years); 57.5% had commercial insurance, 12.4% were insured by Medicaid, and 28.7% were insured by Medicare; 35.8% were Black, 53% were White, and 4.0% were Hispanic or Latino; 84.1% were active electronic patient portal users (Table 1). The intervention was conducted May 27, 2022, to August 5, 2022, when all outreach was complete. Each patient was followed for a total of 6 months.

### FIT Completion

At 4 months, 17.8% (95% CI, 16.3%–19.2%) of patients who received the box vs 18.0% (95% CI, 16.5%–19.5%) of those who received the standard envelope completed FIT, an absolute difference of −0.2% (95% CI, −2.3% to 1.9%; *P* = .85) (Table 2). Comparing the text message interventions, 21.2% (95% CI, 19.6%–22.8%) of patients who received a text message vs 14.6% (95% CI, 13.2%–15.9%) of those who did not receive a text reminder completed FIT, an absolute difference of 6.6% (95% CI, 4.6%–8.7%; *P* < .001). The number needed to outreach (1/absolute difference in response rate) for the text reminder at 4 months is 15.2. Similarly comparing the mailed reminder arms, 20.3% (95% CI, 18.7%–21.8%) of patients who received the mailed reminder vs 15.5% (95% CI, 14.1%–16.9%) of those who did not receive the reminder completed FIT, an absolute difference of 4.8% (95% CI, 2.7%–6.9%; *P* < .001). The number needed to outreach for the mailed reminder at 4 months is 20.8. At 6 months, there were similar results for all 3 comparisons (Table 2). Response rates for each of the 8 randomized groups are seen in Supplementary Table 2.

### CRC Screening Completion

At 4 months, 21.4% (95% CI, 19.8%–22.9%) of patients who received the branded box completed some form of screening, compared with 21.8% (95% CI, 20.2%–23.4%) in the standard envelope group, an absolute difference of −0.5% (95% CI, −2.7 to 1.8%; *P* = .69) (Table 2). Among those receiving text messaging, 24.7% (95% CI, 23.0%–26.3%) of patients had completed some form of screening at 4 months, vs 18.5% (95% CI, 17.0%–20.0%) of those who did not receive text messaging, a difference of 6.2% (95% CI, 4.0%–8.5%; *P* < .001). Comparing the mailed reminder arms, 23.9% (95% CI, 22.2%–25.5%) of patients who received the mailed reminder vs 19.3% (95% CI, 17.8%–20.8%) of those who did not receive the reminder completed FIT, an absolute difference of 4.6% (95% CI, 2.4%–6.8%; *P* < .001). There were similar results for CRC screening completion at 6 months (Table 2).

### CRC Screening Completion at 4 Months vs Nontrial Comparator Groups

Of the 5244 trial participants receiving the intervention (All RCT cohort), 1132 (21.6%; 95% CI, 20.5%–22.7%) completed any CRC screening, compared with 208 of 3765 (5.5%; 95% CI, 4.8%–6.3%) patients not in the clinical trial (All Comparator cohort), an absolute difference of 16.1% (95% CI, 14.7%–17.4%; *P* < .001). Of the trial participants from the split practice, 133 of 506 (26.3%; 95% CI, 22.4%–30.1%) completed CRC screening (Bucks RCT cohort), compared with 42 of 514 (8.2%; 95% CI, 5.8%–10.5%) of the eligible and non-participating patients from this practice (Bucks Comparator cohort), an absolute difference of 18.1% (95% CI, 13.6%–22.6%, *P* < .001) (Table 3).

### Screening Outcomes and Follow-up

At 4 months from initial outreach, 938 of 5244 patients (17.9%) completed FIT across all trial arms. Of those, 46 (4.9%) resulted as positive and 28 (61.0%) completed follow-up colonoscopy within 8 months of their positive result. Follow-up rates were similar across arms, ranging from a low of 53.8% to a high of 70.0% (Supplementary Table 3).

Among the 28 who completed follow-up, 15 (53.6%) had at least one adenoma, and at least 28 adenomas were removed (1 patient had no pathology available and 2 had only follow-up encounters mentioning adenomatous polyps without further specification). This group also included a patient with a 15-mm adenomatous polyp with focal high-grade dysplasia, one with a 15-mm neuroendocrine tumor in the terminal ileum, and a new diagnosis of ulcerative colitis.

### Subgroup Analyses

We did not find heterogeneous treatment effects for any of the interventions in the subgroup analyses when accounting for multiple comparisons (Supplementary Tables 4–6, Supplementary Figures 1–3).

## Discussion

In this pragmatic trial, we found that text messaging and mailed reminders independently increased response to mailed FIT outreach by 6.7 and 4.8 percentage points, respectively, but a prominent health system-branded box did not have a statistically significant effect. Additionally, we found that mailed FIT outreach overall resulted in greater CRC screening of any form than usual care among primary care practices at an academic health system. The effect size of a 16 percentage point increase in CRC screening in the mailed FIT cohort is comparable to what was found in a meta-analysis of prior RCTs.^25^

There are a few behavioral reasons why the text messaging and mailed reminders increased effectiveness of outreach. First, it reminded patients about returning the kit and the reasons to complete screening. Second, the messaging invokes social norms as it highlights that the primary care clinician has recommended screening for the patient.^16,17^ The patient already has a relationship with the clinician so there is trust as well as the endorsement that this is beneficial for the patient’s health. The name of the clinician is explicitly mentioned in the text messaging as well as the signature of the reminder letter. Another social norm is reciprocity, and the messages describe that the health system has sent the patients and kit and paid for postage, so patients may feel compelled to return the kit.^18^ The texting also allows patients to commit to completing the screening by texting back ‘plan,’ which acts as an implementation intention. Finally, text messaging provides an easy mechanism for patients to ask questions about the test and communicate back that they have already sent back the kit or completed colonoscopy.

For both text messaging and the reminder letter, we also found similar response rates by most sociodemographic factors. In the case of text messaging, Black patients had a similar effect size to other racial groups, which is important becaise there are disparities in outcomes for CRC, and there has been mixed evidence of effectiveness of text messaging in preventive health outreach.^28–30^ Text messaging does not require home internet or a smartphone, so it may be more accessible than the electronic patient portal.

This study is consistent with prior trials that have shown increase in uptake from text and mailed reminders.^7^ A meta-analysis showed that reminders from different modalities (mailed, automated, or live reminders) increased response rate to CRC screening outreach by about 3 percentage points, whereas live phone calls had a 6 percentage point effective size.^25^ Another trial evaluating mailed FIT outreach at a community health setting in 2016 showed that 16.9% of patients returned FIT at 6 months when receiving text reminders compared with 23.7% among those that received mailed reminders.^31^ A randomized study from the VA Puget Sound Health Care System in 2021 showed a 10 percentage point increase in response from text messaging.^32^ The greater response rate to texting compared with mailed letter in our study suggests that there may be more access to text messaging among patients, which reflects broader trends in technology use.

Why did the health system-branded box not have greater response rate compared with the standard envelope? Pilot data at our health system showed promise in this approach, which aligns with the behavioral intervention of reciprocity while being more noticeable and attractive to patients. One possibility is that mailing the kits to patients’ homes along with refinements in language to the outreach letter may already be sufficient in making the outreach noticeable and easy to use. This is consistent with a study of specialized packaging for malaria treatment, which did not show effectiveness.^33^ This also highlights the value and importance of randomized and pragmatic evaluations for health system interventions that show promise and require additional resources. The box costs about $4 more per kit to produce than the standard envelope, so this is an important question for efficiency and scale in a limited resource setting.

The strengths of this study are the pragmatic design conducted in the context of real-world clinical operations for a large urban health system. We rigorously evaluated interventions that showed both promise but also have costs for implementation, and we incorporated the latest insights from behavioral science. The population was also diverse with 36% Black patients, who are known to have disparities in CRC screening and outcomes.

There are some limitations. The results of this academic health system may not translate directly to other populations. For example, colonoscopy is still predominantly used and encouraged by clinicians, which may have limited response rates. Additionally, the patients included are those that are not up-to-date despite prior campaigns and health system efforts, so they may have more hesitation about screening and outreach. Further, statistical analyses did not adjust for the randomization factor (site) and so CIs may have been conservative.

## Conclusion

In conclusion, we find that text and mailed reminders can significantly increase mailed FIT outreach response rates, particularly when embedded with behavioral science principles. Pragmatic trials embedded in operations can both evaluate interventions in the context of important clinical programs for cancer screening. More intensive efforts such as phone calls or navigation that include different choices of testing may be needed to substantially increase screening rates.

## Supplementary Material

1

Note: To access the supplementary material accompanying this article, visit the online version of *Clinical Gastroenterology and Hepatology* at www.cghjournal.org, and at https://doi.org/10.1016/j.cgh.2025.09.015.

## Figures and Tables

**Table 1.** Demographics

	Arm 1 Box/text/remind	Arm 2 Box/text no remind	Arm 3 Box/no text/remind	Arm 4 Box/no text/no remind	Arm 5 Env/text/remind	Arm 6 Env/text/no Remind	Arm 7 Env/no text/remind	Arm 8 Env/no text/no remind	Total
n	656	661	653	661	655	656	653	649	5244
Age, *years*	59.3 (7.2)	60.0 (7.4)	59.7 (7.4)	59.6 (7.5)	59.8 (7.3)	59.1 (7.1)	59.9 (7.3)	59.5 (7.2)	59.6 (7.3)
Sex									
Female	376 (57.3)	377 (57.0)	391 (59.9)	388 (58.7)	366 (55.9)	352 (53.7)	375 (57.4)	371 (57.2)	2,996 (57.1)
Male	280 (42.7)	284 (43.0)	262 (40.1)	273 (41.3)	289 (44.1)	304 (46.3)	278 (42.6)	278 (42.8)	2,248 (42.9)
Race									
Asian	30 (4.6)	27 (4.1)	35 (5.4)	22 (3.3)	29 (4.4)	26 (4.0)	26 (4.0)	25 (3.9)	220 (4.2)
Black	226 (34.5)	221 (33.4)	241 (36.9)	242 (36.6)	239 (36.5)	238 (36.3)	242 (37.1)	229 (35.3)	1,878 (35.8)
White	357 (54.4)	369 (55.8)	342 (52.4)	342 (51.7)	345 (52.7)	332 (50.6)	349 (53.5)	344 (53.0)	2,780 (53.0)
Other^a^	17 (2.6)	19 (2.9)	22 (3.4)	29 (4.4)	22 (3.4)	34 (5.2)	16 (2.5)	27 (4.2)	186 (3.5)
Unknown/refused	26 (4.0)	25 (3.8)	13 (2.0)	26 (3.9)	20 (3.1)	26 (4.0)	20 (3.1)	24 (3.7)	180 (3.4)
Ethnicity									
Hispanic/Latino	23 (3.5)	30 (4.5)	21 (3.2)	32 (4.8)	32 (4.9)	28 (4.3)	27 (4.1)	15 (2.3)	208 (4.0)
Non-Hispanic/Latino	619 (94.4)	620 (93.8)	619 (94.8)	616 (93.2)	601 (91.8)	619 (94.4)	616 (94.3)	615 (94.8)	4,925 (93.9)
Unknown/refused	14 (2.0)	11 (1.7)	13 (2.0)	13 (2.0)	22 (3.4)	9 (1.4)	10 (1.5)	19 (2.9)	111 (2.1)
Patient portal status									
Active	549 (83.7)	555 (84.0)	544 (83.3)	557 (84.3)	553 (84.4)	556 (84.8)	555 (85.0)	542 (83.5)	4,411 (84.1)
Not active	107 (16.3)	106 (16.0)	109 (16.7)	104 (15.7)	102 (15.6)	100 (15.2)	98 (15.0)	107 (16.5)	833 (15.9)
Prior screening (any)	188 (28.7)	195 (29.5)	212 (32.5)	192 (29.1)	194 (29.6)	187 (28.5)	210 (32.2)	178 (27.4)	1,556 (29.7)
Insurance type									
Medicaid	77 (11.8)	71 (10.8)	73 (11.3)	80 (12.2)	84 (12.9)	84 (12.9)	91 (14.1)	84 (13.1)	644 (12.4)
Medicare	175 (26.9)	208 (31.7)	172 (26.5)	197 (30.0)	191 (29.3)	186 (28.6)	185 (28.7)	177 (27.5)	1,491 (28.7)
Commercial	388 (59.6)	371 (56.6)	392 (60.5)	369 (56.2)	369 (56.5)	371 (57.0)	360 (55.9)	373 (58.0)	2,993 (57.5)
Unknown	11 (1.7)	6 (0.9)	11 (1.7)	11 (1.7)	9 (1.4)	10 (1.5)	8 (1.2)	9 (1.4)	75 (1.4)
Median household income^b,c^	$76,973 ($39,672 – 104,232)	$77,091 ($46,698 – 104,232)	$77,091 ($46,698 – 104,232)	$76,854 ($39,672 – 104,232)	$74,349 ($39,672 – 104,232)	$77,091 ($39,672 – 104,232)	$76,610 ($43,556 – 104,232)	$73,762 ($39,672 – 104,232)	$76,875 ($41,523 – 104,232)

NOTE. Data are presented as number (%), mean (standard deviation), or median (interquartile range).

Env, envelope.

a Other includes Native Hawaiian/Pacific Islander (15), American Indian/Alaska Native (21), as well as those who self-identified as other (150).

b Fifty-three participants missing median income data.

c Income data from American Community Survey (ACS) 2017–2021 (5-year estimates).

**Table 2.** Screening Completion Within 4 and 6 Months by Combined Study Arm

Study arm	N	4 months			Number needed to outreach^b^	6 months		
		FIT completion, n (%) [95% CI]	Difference, % [95% CI]	*P* value		FIT completion, n (%) [95% CI]	Difference, % [95% CI]	*P* value
Box (Arms 1+2+3+4)	2631	468 (17.8) [16.3 – 19.2]	−0.2 [−2.3 to 1.9]	.85	–	505 (19.2) [17.7 to 20.7]	0.4 [−1.8 to 2.5]	.74
Envelope (Arms 5+6+7+8)	2613	470 (18.0) [16.5 – 19.5]				492 (18.8) [17.3 to 20.3]		
Texting (Arms 1+2+5+6)	2628	557 (21.2) [19.6 to 22.8]	6.6 [4.6 to 8.7]	< .001	15.2	592 (22.5) [20.9 to 24.1]	7.0 [4.9 to 9.2]	< .001
No texting (Arms 3+4+7+8)	2616	381 (14.6) [12.2 – 15.9]				405 (15.5) [14.1 to 16.9]		
Reminder (Arms 1+3+5+7)	2617	531 (20.30) [18.7 to 21.8]	4.8 [2.7 to 6.9]	< .001	20.8	558 (21.3) [19.8 to 22.9]	4.6 [2.5 to 6.7]	< .001
No reminder (Arms 2+4+6+8)	2627	407 (15.5) [14.1 to 16.9]				439 (16.7) [15.3 to 18.1]		

Study arm	N	Any completion, ^a^ n (%) [95% CI]	Difference, (95% CI)	*P* value	Number needed to outreach^b^	Any completion,^a^ n (%) [95% CI]	Difference, % (95% CI)	*P* value

Box (Arms 1+2+3+4)	2631	562 (21.4) [19.8–22.9]	− 0.5 [−2.7 to 1.8]	.69	–	650 (24.7) [23.1–26.4]	0.2 [−2.5 to 2.6]	.86
Envelope (Arms 5+6+7+8)	2613	570 (21.8) [20.2–23.4]				640 (24.5) [22.8–26.1]		
Texting (Arms 1+2+5+6)	2628	649 (24.7) [23.0–26.3]	6.2 [4.0–8.5]	< .001	16.1	739 (28.1) [26.4–29.8]	7.1 [4.7–9.4]	< .001
No texting (Arms 3+4+7+8)	2616	483 (18.5) [17.0–20.0]				551 (21.1) [19.5–22.6]		
Reminder (Arms 1+3+5+7)	2617	625 (23.9) [22.2–25.5]	4.6 [2.4–6.8]	< .001	21.7	700 (26.7) [25.1–28.4]	4.3 [2.0–6.6]	< .001
No reminder (Arms 2+4+6+8)	2627	507 (19.3) [17.8–20.8]				590 (22.5) [20.9–24.1]		

CI, confidence interval,.

a Includes FIT, FOBT, Cologuard, or colonoscopy completion.

b Number needed to outreach = 1/absolute difference in response rate.

**Table 3.** Screening Completion in 4 Months by RCT vs Comparator Groups

Study arm	N	FIT completion, n (%) [95% CI]	Difference, % (95% CI)	*P* value	Any completion,^a^ n (%) [95% CI]	Difference, % (95% CI)	*P* value
All RCT cohort	5244	938 (17.9) [16.8–18.9]	16.9 (15.8–18.0)	<.001	1,132 (21.6) [20.5–22.7]	16.1 (14.7–17.4)	<.001
All Comparator cohort	3765	37 (1.0) [0.7–1.3]			208 (5.5) [4.8–6.3]		
Bucks RCT cohort	506	108 (21.3) [17.8–24.9]	20.8 (17.1–24.4)	<.001	133 (26.3) [22.4–30.1]	18.1 (13.6–22.6)	<.001
Bucks Comparator cohort	514	3 (0.6) [−0.1 to 1.2]			42 (8.2) [5.8–10.5]		

CI, confidence interval; RCT, randomized controlled trial.

a Includes FIT, FOBT, Cologuard, or colonoscopy completion.

## Data Availability

Outreach materials and methods are available in the Protocol Supplement. Otherwise, patient data will not be shared.

## What You Need to Know

### Background

It is unclear if behavioral interventions such as prominent packaging, text messaging, and mailed reminders increase response to mailed colorectal cancer screening outreach.

### Findings

In this pragmatic clinical trial of 5244 patients, we found that text messaging and mailed reminders significantly increased uptake.

### Implications for patient care

Communications modalities such as text messaging and mailed reminders can be used in population health outreach programs to increase cancer screening rates.

## Abbreviations

CI: confidence interval
CONSORT: Consolidated Standards of Reporting Trials
CRC: colorectal cancer
EHR: electronic health record
FIT: fecal immunochemical testing
PCP: primary care provider
RCT: randomized controlled trial
W2H: Way to Health
