# Supplementary material for: Behavioral Interventions Improve Mailed Colorectal Cancer Screening Among Overdue Patients in a Randomized Trial
Source: Clin Gastroenterol Hepatol. Author manuscript; Available in PMC 2026 May 29. (PMC13220170; doi:10.1016/j.cgh.2025.09.015)

Supplemental Materials

Supplement Table 1: Texting engagement……………………………………………………………….2

Supplement Table 2: Screening completion within 4 months by individual study arm …………………...3

Supplement Table 3: Colonoscopy completion after positive FIT by study arm…………………………..4

Supplement Table 4: Subgroup analysis for receiving the box compared to not receiving the box…….….5

Supplement Table 5: Subgroup analysis for receiving texting compared to not receiving texting……..….6

Supplement Table 6: Subgroup analysis for receiving mailed reminders compared to not receiving mailed reminders…………………………………………………………………………………………....7

Supplement Figure 1: Subgroup analysis for receiving the box compared to not receiving the box………8

Supplement Figure 2: Subgroup analysis for receiving texting compared to not receiving texting..…...….9

Supplement Figure 3: Subgroup analysis for receiving mailed reminders compared to not receiving mailed reminders……………………………………………………………………………………..….10

Supplement Table 1. Texting engagement

Patients Receiving Texting, N	Texted DONE, N (%)	Texted MISSING, N (%)	Texted COLO, N (%)	Texted PLAN, N (%)	Total Engagement, N (%)
2,628	190 (7.2)	152 (5.8)	86 (3.3)	9 (0.3)	437 (16.6)

Supplement Table 2. Screening completion within 4 months by individual study arm

	N	Completed, N (%)
**Arm 1** Box/Text/Remind	656	144 (21.6)
**Arm 2** Box/Text/No Remind	661	121 (18.3)
**Arm 3** Box/NoText/Remind	653	135 (20.7)
**Arm 4** Box/No Text/NoRemind	661	68 (10.3)
**Arm 5** Env/Text/Remind	655	154 (23.5)
**Arm 6** Env/Text/No Remind	656	138 (21.0)
**Arm 7** Env/NoText/Remind	653	98 (15.0)
**Arm 8** Env/No Text/ NoRemind	649	80 (12.3)
**Total**	5,244	938 (17.9)

Supplement Table 3. Colonoscopy completion after positive FIT by study arm

	Positive FIT, N	Colonoscopy, N (%)
**Box**<br>(Arms 1+2+3+4)	19	13 (68.4)
**Envelope**<br>(Arms 5+6+7+8)	27	15 (55.6)
**Texting**<br>(Arms 1+2+5+6)	35	20 (57.1)
**No Texting**<br>(Arms 3+4+7+8)	11	8 (72.7)
**Reminder**<br>(Arms 1+3+5+7)	26	14 (53.8)
**No Reminder**<br>(Arms 2+4+6+8)	20	14 (70.0)

Supplement Table 4. Subgroup analysis for receiving the box compared to not receiving the box (n=5,170)

**Subgroup**	**Odds Ratio (95% Confidence Interval) for FIT completion**	**P-value**	**P-value for interaction**
All patients	0.98 (0.85, 1.14)	0.82	
**Received Texting**			0.06
No	1.15 (0.92, 1.44)	0.21	
Yes	0.87 (0.72, 1.06)	0.16	
**Received Mailed Reminder**			0.15
No	0.92 (0.74, 1.13)	0.42	
Yes	1.13 (0.93, 1.37)	0.22	
**Sex**			0.28
Female (n=2,996)	0.96 (0.80, 1.16)	0.68	
Male (n=2,248)	1.13 (0.90, 1.42)	0.28	
**Age**			0.31
**Race**			0.04
White (n=2,780)	1.15 (0.95, 1.39)	0.16	
Asian (n=220)	0.55 (0.29, 1.03)	0.06	
Black (n=1,878)	0.95 (0.73, 1.23)	0.67	
Other (n=366)	1.72 (0.78, 3.80)	0.18	
**Ethnicity**			0.26
Not Hispanic or Latino (n=4,925)	1.03 (0.89, 1.20)	0.69	
Hispanic or Latino (n=208)	1.44 (0.68, 3.07)	0.34	
Other/Unknown (n=111)	0.52 (0.20, 1.39)	0.19	
**Income**			0.58
**Insurance**			0.68
Commercial (n=2,993)	1.07 (0.89, 1.29)	0.47	
Medicaid (n=644)	0.85 (0.49, 1.48)	0.56	
Medicare (n=1,491)	1.01 (0.78, 1.30)	0.95	
Other/Unknown (n=75)	0.51 (0.11, 2.34)	0.38	
**Patient Portal Status**			0.15
Not Activated (n=833)	1.40 (0.90, 2.20)	0.14	
Activated (n=4,411)	0.99 (0.85, 1.15)	0.90	
**Any prior CRC screening**			0.44
No (n=3,671)	1.07 (0.89, 1.30)	0.44	
Yes (n=1,573)	0.96 (0.77, 1.20)	0.73	

Supplement Table 5. Subgroup analysis for receiving texting compared to not receiving texting (n=5,170)

Subgroup	Odds Ratio (95% Confidence Interval) for FIT completion	P-value	P-value for interaction
All patients	1.61 (1.39, 1.87)	<0.001	
**Received the Box**			0.06
No	1.86 (1.50, 2.29)	<0.001	
Yes	1.40 (1.14, 1.72)	0.001	
**Received the Mailed Reminder**			0.06
No	1.89 (1.53, 2.36)	<0.001	
Yes	1.43 (1.17, 1.73)	<0.001	
**Sex**			0.06
Female (n=2954)	1.45 (1.20, 1.75)	<0.001	
Male (n=2216)	1.93 (1.53, 2.43)	<0.001	
**Age**			0.17
**Race**			0.55
White (n=2745)	1.49 (1.23, 1.80)	<0.001	
Asian (n=216)	1.48 (0.79, 2.76)	0.22	
Black (n=1849)	1.80 (1.38, 2.35)	<0.001	
Other (n=360)	2.65 (1.15, 6.09)	0.02	
**Ethnicity**			0.96
Not Hispanic or Latino (n=4854)	1.62 (1.39, 1.88)	<0.001	
Hispanic or Latino (n=207)	1.65 (0.77, 3.54)	0.20	
Other/Unknown (n=109)	1.85 (0.72, 4.76)	0.21	
**Income**			0.82
**Insurance**			0.87
Commercial (n=2975)	1.57 (1.30, 1.89)	<0.001	
Medicare (n=1479)	1.76 (1.36, 2.28)	<0.001	
Medicaid (n=641)	1.56 (0.89, 2.71)	0.12	
Other/Unknown (n=75)	1.20 (0.27, 5.29)	0.81	
**Patient Portal Status**			0.72
Not Activated (n=817)	1.76 (1.12, 2.76)	0.02	
Activated (n=4353)	1.61 (1.38, 1.87)	<0.001	
**Any prior CRC screening**			0.09
No (n=3629)	1.46 (1.21, 1.76)	<0.001	
Yes (n=1541)	1.88 (1.50, 2.35)	<0.001	

Supplement Table 6. Subgroup analysis for receiving mailed reminders compared to not receiving mailed reminders (n=5,170)

Subgroup	Odds Ratio (95% Confidence Interval) for FIT completion	P-value	P-value for interaction
All patients	1.40 (1.21, 1.62)	<0.001	
**Received the Box**			0.16
No	1.23 (1.00, 1.50)	0.05	
Yes	1.51 (1.23, 1.85)	<0.001	
**Received Text Messaging**			0.06
No	1.60 (1.29, 2.00)	<0.001	
Yes	1.21 (1.00, 1.46)	0.05	
**Sex**			0.55
Female (n=2954)	1.41 (1.17, 1.70)	<0.001	
Male (n=2216)	1.29 (1.03, 1.62)	0.03	
**Age**			0.92
**Race**			0.06
White (n=2745)	1.48 (1.22, 1.79)	<0.001	
Black (n=1849)	1.21 (0.93, 1.57)	0.15	
Asian (n=216)	0.85 (0.46, 1.59)	0.62	
Other/Unknown (n=360)	3.25 (1.43, 7.38)	0.005	
**Ethnicity**			0.59
Not Hispanic or Latino (n=4854)	1.36 (1.17, 1.58)	<0.001	
Hispanic or Latino (n=207)	1.10 (0.52, 2.31)	0.81	
Other/Unknown (n=109)	2.07 (0.79, 5.47)	0.14	
**Income**			0.69
**Insurance**			0.91
Commercial (n=2975)	1.41 (1.17, 1.69)	<0.001	
Medicare (n=1479)	1.29 (0.99, 1.66)	0.05	
Medicaid (n=641)	1.41 (0.81, 2.46)	0.23	
Unknown (n=75)	0.96 (0.22, 4.23)	0.96	
**Patient Portal Status**			0.57
Not Activated (n=817)	1.54 (0.98, 2.41)	0.06	
Activated (n=4353)	1.34 (1.15, 1.56)	<0.001	
**Any prior CRC screening**			0.55
No (n=3629)	1.41 (1.17, 1.71)	<0.001	
Yes (n=1541)	1.29 (1.03, 1.62)	0.02	

Supplement Figure 1. Subgroup analysis for receiving the box compared to not receiving the box

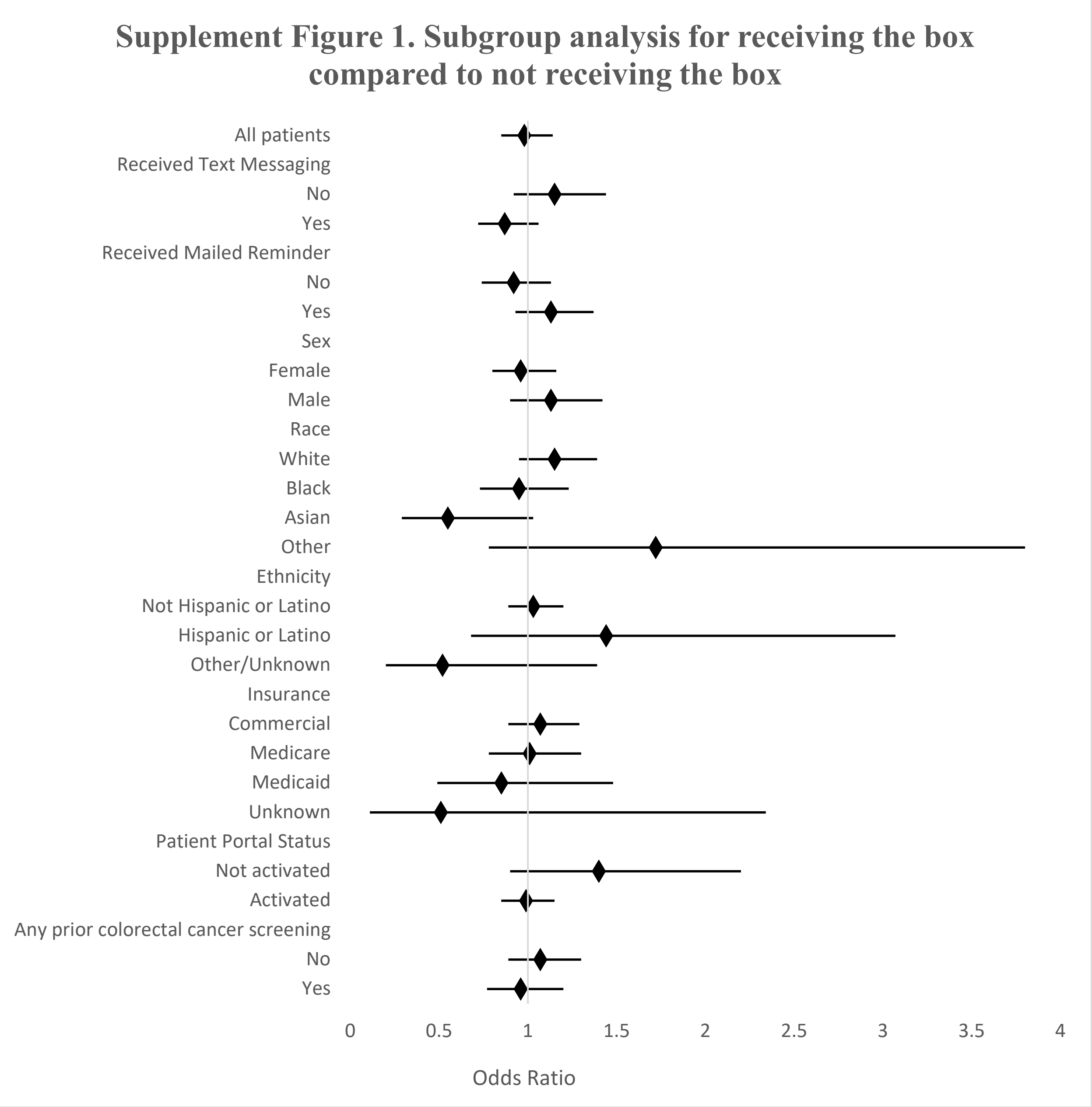

Supplement Figure 2. Subgroup analysis for receiving texting compared to not receiving texting

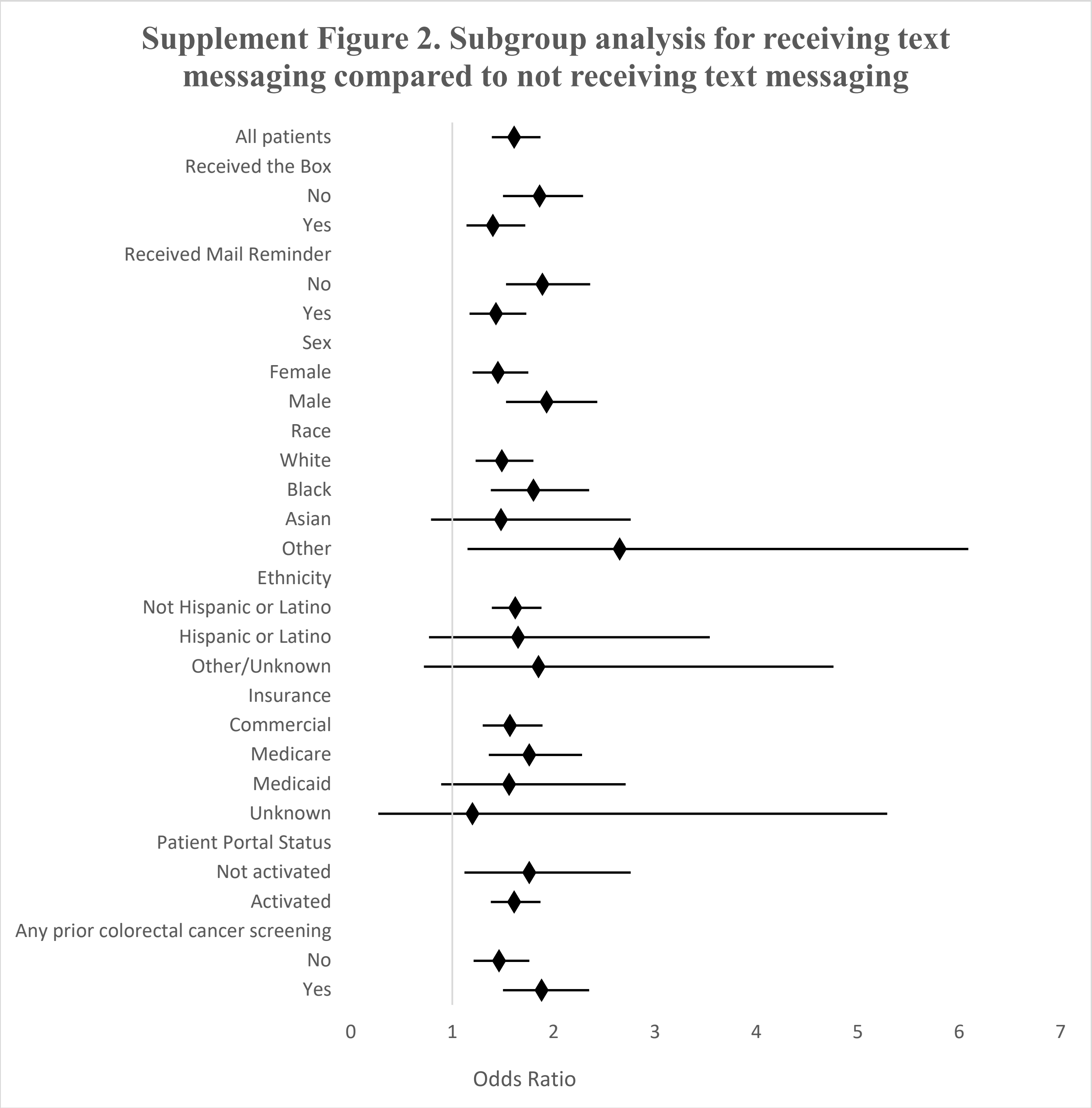

Supplement Figure 3. Subgroup analysis for receiving mailed reminders compared to not receiving mailed reminders

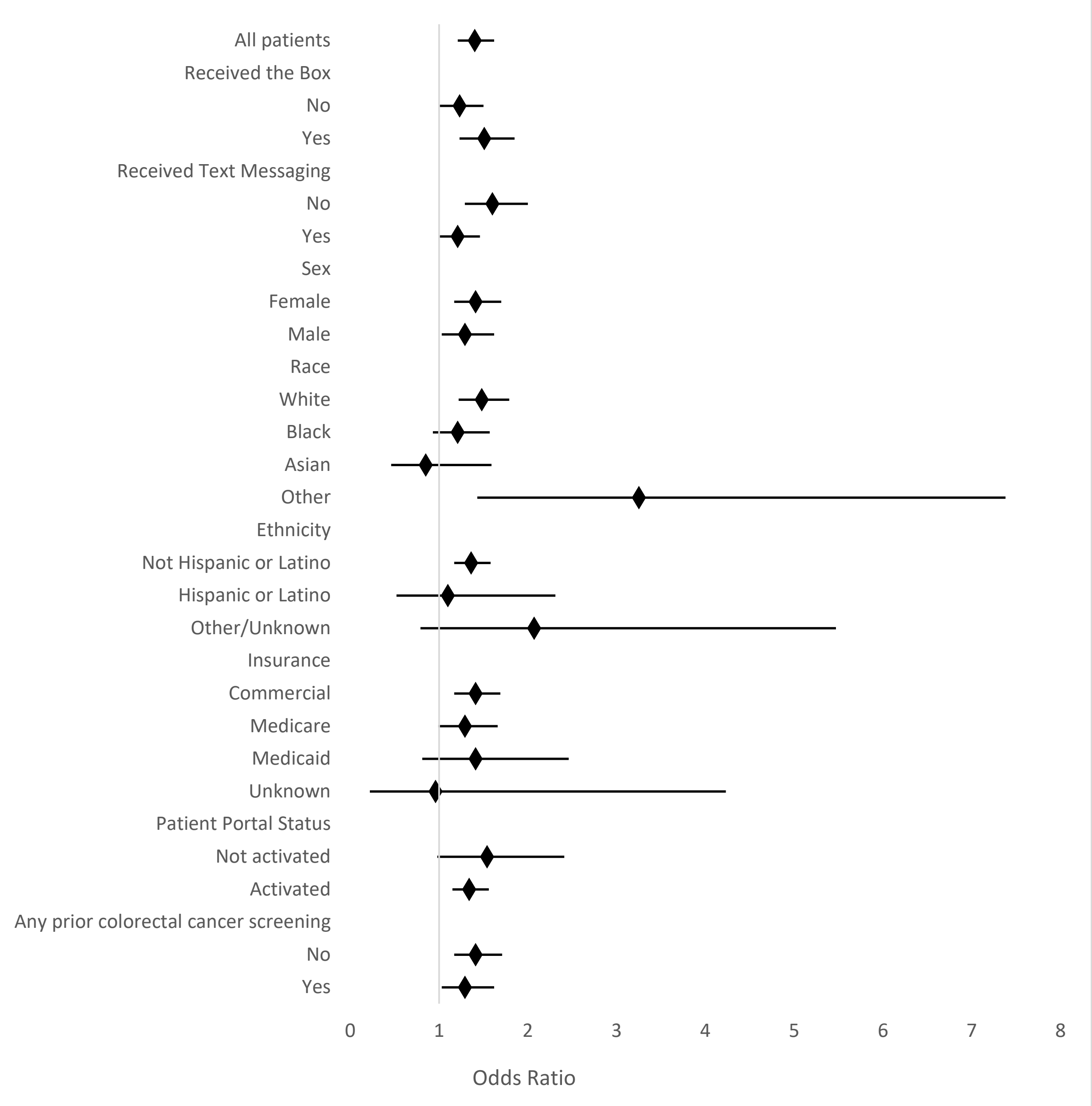

Supplement: 1 [file NIHMS2173289-supplement-1.pdf]
